# Supplementary material for: ADAMTSL2 is a potential prognostic biomarker and immunotherapeutic target for colorectal cancer: Bioinformatic analysis and experimental verification
Source: PLoS One. 2024 May 30;19(5):e0303909. doi: 10.1371/journal.pone.0303909 (PMC11139340; doi:10.1371/journal.pone.0303909)
Supplement: S1 Table — (DOCX) [file pone.0303909.s001.docx]

**S1 Table.** Correlation of ADAMTSL2 expression with clinical features in CRC patients.

| **characteristics** | **Overall** | **Low expression of ADAMTSL2** | **High expression of ADAMTSL2** | **P value** | **statistic** | **method** |
| --- | --- | --- | --- | --- | --- | --- |
| n | 644 | 322 | 322 |  |  |  |
| Pathologic T stage, n (%) |  |  |  | 0.089 | 6.511 | Chisq test |
| T1 | 20 (3.1%) | 10 (1.6%) | 10 (1.6%) |  |  |  |
| T2 | 111 (17.3%) | 56 (8.7%) | 55 (8.6%) |  |  |  |
| T3 | 436 (68%) | 229 (35.7%) | 207 (32.3%) |  |  |  |
| T4 | 74 (11.5%) | 27 (4.2%) | 47 (7.3%) |  |  |  |
| Pathologic N stage, n (%) |  |  |  | 0.000 | 19.429 | Chisq test |
| N0 | 368 (57.5%) | 211 (33%) | 157 (24.5%) |  |  |  |
| N1 | 153 (23.9%) | 67 (10.5%) | 86 (13.4%) |  |  |  |
| N2 | 119 (18.6%) | 43 (6.7%) | 76 (11.9%) |  |  |  |
| Pathologic stage, n (%) |  |  |  | 0.000 | 26.735 | Chisq test |
| Stage I | 111 (17.8%) | 55 (8.8%) | 56 (9%) |  |  |  |
| Stage II | 238 (38.2%) | 148 (23.8%) | 90 (14.4%) |  |  |  |
| Stage III | 184 (29.5%) | 81 (13%) | 103 (16.5%) |  |  |  |
| Stage IV | 90 (14.4%) | 30 (4.8%) | 60 (9.6%) |  |  |  |
| Gender, n (%) |  |  |  | 0.179 | 1.803 | Chisq test |
| Female | 301 (46.7%) | 159 (24.7%) | 142 (22%) |  |  |  |
| Male | 343 (53.3%) | 163 (25.3%) | 180 (28%) |  |  |  |
| Age, n (%) |  |  |  | 0.000 | 21.330 | Chisq test |
| <= 65 | 276 (42.9%) | 109 (16.9%) | 167 (25.9%) |  |  |  |
| > 65 | 368 (57.1%) | 213 (33.1%) | 155 (24.1%) |  |  |  |
| Histological type, n (%) |  |  |  | 0.000 | 19.118 | Chisq test |
| Adenocarcinoma | 550 (86.9%) | 256 (40.4%) | 294 (46.4%) |  |  |  |
| Mucinous adenocarcinoma | 83 (13.1%) | 60 (9.5%) | 23 (3.6%) |  |  |  |
| Neoplasm type, n (%) |  |  |  | 0.001 | 10.519 | Chisq test |
| Colon adenocarcinoma | 478 (74.2%) | 257 (39.9%) | 221 (34.3%) |  |  |  |
| Rectum adenocarcinoma | 166 (25.8%) | 65 (10.1%) | 101 (15.7%) |  |  |  |
