# Supplementary material for: ADAMTSL2 is a potential prognostic biomarker and immunotherapeutic target for colorectal cancer: Bioinformatic analysis and experimental verification
Source: PLoS One. 2024 May 30;19(5):e0303909. doi: 10.1371/journal.pone.0303909 (PMC11139340; doi:10.1371/journal.pone.0303909)
Supplement: S2 Table — (DOCX) [file pone.0303909.s002.docx]

**S2 Table**. Correlation between ADAMTSL2 expression and clinical characteristics (logistic analysis).

| **Characteristics** | **Total (N)** | **OR (95% CI)** | **P value** |
| --- | --- | --- | --- |
| Pathologic T stage (T3&T4 vs. T1&T2) | 641 | 1.007 (0.686 - 1.479) | 0.97 |
| Pathologic N stage (N1&N2 vs. N0) | 640 | 1.979 (1.440 - 2.721) | < 0.001 |
| Pathologic stage (Stage III&Stage IV vs. Stage I&Stage II) | 623 | 2.042 (1.480 - 2.816) | < 0.001 |
| Gender (Male vs. Female) | 644 | 1.236 (0.907 - 1.686) | 0.18 |
| Age (> 65 vs. <= 65) | 644 | 0.475 (0.346 - 0.653) | < 0.001 |
| Histological type (Mucinous adenocarcinoma vs. Adenocarcinoma) | 633 | 0.334 (0.201 - 0.555) | < 0.001 |
| Neoplasm type (Rectum adenocarcinoma vs. Colon adenocarcinoma) | 644 | 1.807 (1.261 - 2.589) | 0.001 |
