# Supplementary material for: ADAMTSL2 is a potential prognostic biomarker and immunotherapeutic target for colorectal cancer: Bioinformatic analysis and experimental verification
Source: PLoS One. 2024 May 30;19(5):e0303909. doi: 10.1371/journal.pone.0303909 (PMC11139340; doi:10.1371/journal.pone.0303909)
Supplement: S3 Table — (DOCX) [file pone.0303909.s003.docx]

**S3 Table**. Univariate and multivariate analysis of OS and clinical characteristics (Cox regression).

| **Characteristics** | **Total (N)** | **Univariate analysis** | | **Multivariate analysis** | |
| --- | --- | --- | --- | --- | --- |
|  |  | **HR(95% CI)** | **P value** | **HR(95% CI)** | **P value** |
| Pathologic T stage (T1&T2 vs. T3&T4) | 640 | 2.468 (1.327 - 4.589) | 0.004 | 1.828 (0.933 - 3.583) | 0.079 |
| Pathologic N stage (N0 vs. N1&N2) | 639 | 2.627 (1.831 - 3.769) | < 0.001 | 0.273 (0.108 - 0.690) | 0.006 |
| Pathologic stage (Stage I&Stage II vs. Stage III&Stage IV) | 622 | 2.988 (2.042 - 4.372) | < 0.001 | 9.740 (3.754 - 25.272) | < 0.001 |
| Gender (Female vs. Male) | 643 | 1.054 (0.744 - 1.491) | 0.769 |  |  |
| Age (<= 65 vs. > 65) | 643 | 1.939 (1.320 - 2.849) | < 0.001 | 2.749 (1.810 - 4.175) | < 0.001 |
| Histological type (Adenocarcinoma vs. Mucinous adenocarcinoma) | 632 | 1.320 (0.810 - 2.151) | 0.266 |  |  |
| Neoplasm type (Colon adenocarcinoma vs. Rectum adenocarcinoma) | 643 | 0.799 (0.519 - 1.230) | 0.308 |  |  |
| ADAMTSL2 (Low vs. High) | 643 | 1.673 (1.176 - 2.381) | 0.004 | 1.654 (1.135 - 2.410) | 0.009 |
