# Supplementary material for: ADAMTSL2 is a potential prognostic biomarker and immunotherapeutic target for colorectal cancer: Bioinformatic analysis and experimental verification
Source: PLoS One. 2024 May 30;19(5):e0303909. doi: 10.1371/journal.pone.0303909 (PMC11139340; doi:10.1371/journal.pone.0303909)
Supplement: Attachment — Submitted filename: Response to reviewers.docx [file pone.0303909.s004.docx]

Dear Editor:

We have carefully studied the valuable comments from reviewers and tried our best to revise the manuscript titled “Identification of ADAMTSL2 as a potential prognosis biomarker associated with immune infiltrates and drug sensitivity in colorectal cancer” (PONE-D-23-31014). The amendment has been marked with red. We have made detailed changes throughout the text. The point to point responds to the reviewer’s comments are listed in the revised text.

**Reviewer #1:**

In the current manuscript the authors analyzed the prognostic roles of ADAMTSL2 in colorectal cancer.

below are my comment about this manuscript

1.The title of the manuscript is unclear. Please add detailed information about the patient group involved in the analysis. For example, include details about tumor stage and whether patients received any treatment before the analysis.

**Response**: Thanks for your comments. The title was improved according to your suggestion.

Abstract

1.The background is insufficient, and the study objective is not precisely described.

**Response**: Thanks for your comments. The background of the abstract was improved according to your suggestion.

2.The study method is unclear; please provide a more detailed description.

**Response**: Thanks for your comments. The abstract methods was improved according to your suggestion.

3.The results are poorly written, and some are confusing, such as the statement, "ADAMTSL2 was associated with ECM-receptor interaction, basal cell carcinoma, dilated cardiomyopathy, TGF-beta signaling pathway, melanoma, arrhythmogenic right ventricular cardiomyopathy (ARVC), Olfactory transduction, complement and coagulation cascades, and hypertrophic cardiomyopathy (HCM)." Please summarize only the results directly related to the study objective.

**Response**: Thanks for your comments. The abstract results were improved according to your suggestion.

4.The conclusion needs clarification.

**Response**: Thanks for your comments. The conclusions were improved according to your suggestion.

Introduction and Method Section:

1.In section 2.5, subgroups were classified into two categories, 0-50 and 50-100. Please specify the criteria used to divide patients into these subgroups.

**Response**: Thanks for your comments. Section 2.5 was improved according to your suggestion.

2.In section 2.7, the second paragraph lacks clarity and does not describe the method used to analyze the correlation between ADAMTSL2 and immune-related genes. Additionally, in section 2.8, the analysis method used to examine the correlation of ADAMTSL2 with TMB/MSI is not described.

**Response**: Thanks for your comments. Sections 2.7 and 2.8 were improved according to your suggestion.

3.Provide more explanation about the analysis used in section 2.9.

**Response**: Thanks for your comments. Section 2.9 was improved according to your suggestion.

4.Clarify section 2.10 in more detail.

**Response**: Thanks for your comments. Section 2.10 was improved according to your suggestion.

Results:

1.In section 3.1, the heading does not describe the findings clearly. The phrase "The results suggested that ADAMTSL2 was associated with tumorigenesis" needs clarification based on the data supporting this conclusion. Another conclusion, "indicating that ADAMTSL2 could be a new biomarker to differentiate CRC from non-tumor tissues," lacks sufficient data for such a claim.

**Response**: Thanks for your comments. The description was improved according to your suggestion.

2.In section 3.2, the description of the results in Figure 2 is not entirely correct and lacks clarity. Please rewrite this section.

**Response**: Thanks for your comments. Section 3.2 was improved according to your suggestion.

3.In section 3.3, the results description is unclear and not entirely accurate. Additionally, there is a discrepancy between your above reported results that ADAMTSL2 is significantly low-expressed in CRC compared with normal colorectal tissue and in this section you stating that patients with high expression of ADAMTSL2 have significantly poor OS and DSS. This contradiction needs clarification.

**Response**: Thanks for your comments. We checked the whole text carefully and Section 3.1 was corrected according to your suggestion.

4.In section 3.4, concentrate the analysis on pathways related to CRC and ignore unrelated pathways.

**Response**: Thanks for your comments. Section 3.4 was improved according to your suggestion.

5.In section 3.5, explain the meaning of READ and COAD patients.

**Response**: Thanks for your comments. The meaning of READ and COAD patients was supplemented in Section 2.1 according to your suggestion.

6.Clarify the main results in Sections 3.6, 3.7, and 3.8.

**Response**: Thanks for your comments. Sections 3.6, 3.7, and 3.8 were improved according to your suggestion.

7.Rewrite section 3.9 for better clarity.

**Response**: Thanks for your comments. Section 3.9 was improved according to your suggestion.

8.The results in section 3.11 contradict those in section 3.1. The expression of ADAMTSL2 is reported as lower in CRC compared to normal tissue in section 3.1, while section 3.11 you reports that ADAMTSL2 is highly expressed in CRC tissues. This discrepancy is unacceptable and requires attention.

**Response**: Thanks for your comments. We checked the whole text carefully and the Section 3.1 was corrected according to your suggestion.

**Reviewer #2:**

In this study, Dr. Huang and their colleagues conducted a comprehensive analysis to evaluate the potential prognostic significance of the ADAMTS-like-2 (ADAMTSL2), as well as the correlation between ADAMTSL2 expression and immune infiltrates as well as drug sensitivity in colorectal cancer. Furthermore, they validated the elevated expression of ADAMTSL2 in colorectal cancer cell lines compared to normal cell line by Real-Time PCR. While the effort and perspective of this paper is interesting, there are several concerns and major revisions are expected.

1. In the introduction section, authors stated “The members of ADAMTSL (a disintegrin-like and metalloproteinase domain with thrombospondin type 1 motif-like) family that secrete glycoproteins are similar to ADAMTS proteases in structure”. This family members secrete glycoproteins? Or this family is kind of secreted glycoproteins. Please rewrite this sentence clearly.

**Response**: Thanks for your comments. The information was improved according to your suggestion.

2. Which database or algorithm was used to evaluate the relationship between ADAMTSL2 and TMB/MSI?

**Response**: Thanks for your comments. The information was supplemented according to your suggestion.

3. Why did the authors include references for some parts of material method section that, in my opinion, it seems unnecessary (such as Ref [32]).

**Response**: Thanks for your comments. The references were improved according to your suggestion.

4. In the result section, 3.1. ADAMTSL2 is aberrantly expressed in pan cancer and CRC part, I think the authors have inaccurately written this paragraph. They stated “The expression of ADAMTSL2 were lower in CRC compared to normal tissue (2.670 ± 0.040 vs. 1.678 ± 0.063, p < 0.001) (Figure 1B). The expression of ADAMTSL2 were lower in CRC tissues compared to paired normal tissues” . But I think ADAMTSL2 expression is higher in CRC compared to normal tissue.

**Response**: Thanks for your comments. We checked the whole text carefully and the section 3.1 was corrected according to your suggestion.

5. In the result section, 3.3. Association of ADAMTSL2 with survival in patients with CRC part, Table S3 shows the results of univariate and multivariate analyses, not the correlation between ADAMTSL2 expression and clinicopathologial characteristics. I think the authors have inaccurately written some parts of this paragraph (the results of univariate analysis).

**Response**: Thanks for your comments. The description about the result of the univariate analysis was improved according to your suggestion.

6. Authors have stated that ADAMTSL2 is highly expressed in CRC tissues compared with normal colorectal tissues in “3.11. ADAMTSL2 was aberrantly expressed in CRC tissues and cell lines part”. However, they have not specified the characteristics of these tissues. Or which correlation was between ADAMTSL2 expression and clinicopathological features in these tissues.

**Response**: Thanks for your comments. We validated ADAMTSL2 expression in CRC cell lines, so we can’t present the correlation between ADAMTSL2 and clinical characteristics. We mention this limitation in the article. This study is a clinical significance and regulatory network analysis of ADAMTSL2 in CRC using the public databases of TCGA and GEO, and these results must be further validated in real world samples.

7. The discussion part was inadequately written. The authors should interpret the results. For example, how this gene is related to the mentioned signaling pathways? Or how the authors interpret the relationship between ADAMTSL2 expression and TMB/MSI? Or the relationship of the ADAMTSL2 high expression group with higher stromal score?

**Response**: Thanks for your comments. The Discussion section was improved according to your suggestion.

8. In the present study, ADAMTSL2 expression is associated with different immune infiltration, some immune checkpoint genes, and signaling pathways. These are highly interesting results. One possible method would be to use siRNA to downregulate the ADAMTSL2 expression in the CRC cell lines or mouse model, assess the expression of some investigated genes, and confirm by experimental examination.

**Response**: Thanks for your comments. We will actively apply for research funding based on existing research results. Once funding support is obtained, we will conduct more detailed research in the future.

9. The figures presented have poor contrast, including Figures 1-8.

**Response**: Thanks for your comments. Figure 1 was improved according to your suggestion.

10. Abbreviations should be fully described the first time they appear, such as COAD, READ, IGLEC15, IDO1, CD274, HAVCR2, PDCD1, CTLA4, LAG3, and PDCD1LG2, …

**Response**: Thanks for your comments. The abbreviations were improved according to your suggestion.

11. There are some grammatical errors throughout, such as:

ADAMTSL2 were associated with

The occurrence and development of CRC is associated

The expression of ADAMTSL2 were lower in CRC tissues compared to paired normal tissues

**Response**: Thanks for your comments. The whole text was improved according to your suggestion.

Thank you for considering our manuscript.

Yours sincerely,

Zhe Huang
