# Supplementary material for: ADAMTSL2 is a potential prognostic biomarker and immunotherapeutic target for colorectal cancer: Bioinformatic analysis and experimental verification
Source: PLoS One. 2024 May 30;19(5):e0303909. doi: 10.1371/journal.pone.0303909 (PMC11139340; doi:10.1371/journal.pone.0303909)
Supplement: Attachment — Submitted filename: #PONE-D-23-31014R1.docx [file pone.0303909.s005.docx]

Review PLOS ONE manuscript #PONE-D-23-31014R1

Zhe Huang (…) Pengzhu Cai. **ADAMTSL2 is associated with immune infiltration and predicts a poor prognosis in colorectal cancer**

**Comments**

This is an interesting manuscript that explores the impact of ADAMTSL2 as a potential prognostic biomarker in colorectal cancer. By using database, the authors conclude that the high expression levels of ADAMTSL2 is associated with poor prognosis in CRC. Although the dataset is huge und the fidings are impressive, the study lacks experimental validation that do not allow to draw strong conclusions at this point.

1. The analysis of the database in the mauscript are strong and the most conclusions are based on these analysis. Key findings should, however, be validated by other methodology. For instance, while the authors claim that ADAMTSL2 expression is highe in CRC tissues compared to normal and paired normal area, assessments on human samples should be performed.
2. All abbreviations should be fully described when they appear for the first time, such as TGF-beta, ECM-receptor and so on. And once the word is described with full description, it should not be repeated. The authors should completely go through the paper again.
3. Tehre are several sentences containing the phrase “and others”. This is not scientific writing and should be revised.

4. The authors claim that ADAMTSL2 is associated with immune infiltration but this cannot be concluded only with the database analysis. Either the statement in accordance with the title of the manuscript should be reviced or the validation assessments using human samples should be conducted.
